# Age-related strength loss affects non-stepping balance recovery

**DOI:** 10.1371/journal.pone.0210049

**Published:** 2019-01-18

**Authors:** Hoda Koushyar, Kathleen A. Bieryla, Maury A. Nussbaum, Michael L. Madigan

**Affiliations:** 1 Department of Biomedical Engineering and Mechanics, Virginia Tech, Blacksburg, VA, United States of America; 2 Department of Mechanical and Biomedical Engineering, University of Portland, Portland, OR, United States of America; 3 Department of Industrial and System Engineering, Virginia Tech, Blacksburg, VA, United States of America; Tokai University, JAPAN

## Abstract

Aging is associated with a higher risk of falls, and an impaired ability to recover balance after a postural perturbation is an important contributing factor. In turn, this impaired recovery ability likely stems from age-related decrements in lower limb strength. The purpose of this study was to investigate the effects of age-related strength loss on non-stepping balance recovery capability after a perturbation while standing, without constraining movements to the ankle as in prior reports. Two experiments were conducted. In the first, five young adults (ages 20–30) and six community-dwelling older adults (ages 70–80) recovered their balance, without stepping, from a backward displacement of a support surface. Balance recovery capability was quantified as the maximal backward platform displacement that a subject could withstand without stepping. The maximal platform displacement was 27% smaller among the older group (11.8±2.1 cm) vs. the young group (16.2±2.6 cm). In the second experiment, forward dynamic simulations of a two-segment, rigid-body model were used to investigate the effects of manipulating strength in the hip extensors/flexors and ankle plantar flexors/dorsiflexors. In these, typical age-related reductions in strength were included. The model predicted lower maximal platform displacements with age-related reductions only in plantar flexion and hip flexion strength. These findings support the previously reported age-related loss of balance recovery ability, and an important role for plantar flexor strength in this ability.

## Introduction

Aging is associated with a high prevalence of falls and falls-related injuries. In the United States, about 40% of adults aged 65 and older fall each year [1], with an estimated 2.8 million older adults having been treated for unintentional fall-related injuries in 2014 [2]. Given that the number of adults age 65 and older is expected to double from 40 million in 2010 to over 80 million in 2050 [3], the high prevalence of falls among this age group is expected to increase.

Perhaps related to the high prevalence of falls, older adults have greater difficulty recovering balance without stepping after being exposed to a postural perturbation. Mackey and Robinovitch [4] determined the largest static lean angle from which young and older women could recover balance upon release without stepping, using the so-called ankle strategy that involves the entire body above the ankles moving primarily as one rigid segment. Older women exhibited a 36% smaller maximum lean angle (13.1 deg) compared to young women (16.3 deg). The greater difficulty among older adults in maintaining standing balance after a postural perturbation is likely related to age-related strength loss. Barrett and Lichtwark [5] performed a simulation study to investigate the effect of age-related changes in neural, muscular, and tendinous parameters on the maximum lean angle from which balance could be recovered upon release when using the ankle strategy. They reported a 19% decrease in the maximum recoverable lean angle after reducing isometric ankle plantar flexor strength by 20%. Conversely, two simulation studies reported improvements in the maximum lean angle after increasing muscle strength. Robinovitch, Heller [6] reported a 64% increase in maximum lean angle after a 100% increase in maximum ankle moment, while Matrangola and Madigan [7] reported a 15% increase in ankle moment resulted in a one degree increase in maximum lean angle. While these studies provide insight on the importance of ankle plantar flexor strength when responding to a postural perturbation with an ankle strategy, larger perturbations while standing elicit hip movement in addition to ankle movement [8, 9]. Removing the ankle strategy constraint, and allowing movement at the hip, should provide additional realism and ecological validity when investigating how age and muscle strength influence standing balance.

The purpose of this study was to expand upon previous work by assessing the effects of age-related strength loss on non-stepping balance recovery ability after a perturbation, but without constraining recovery movements to an ankle strategy. Two experiments were conducted using abrupt anteroposterior displacements of the floor surface as perturbations. These perturbations aimed to displace the base of support with respect to the body center of mass (e.g., similar to standing on a bus or train while it changes speed). In the first experiment, human subjects were used to quantify age-related differences in non-stepping balance recovery ability. We hypothesized that older adults would exhibit a reduced non-stepping balance recovery ability compared to young adults. In the second experiment, we investigated the relative influence of strength loss at individual joints and exertion directions (flexion or extension) on non-stepping balance recovery ability after a perturbation. Given the clear challenge of manipulating strength at individual joints and exertion direction *in vivo*, forward-dynamic computer simulations were used. We hypothesized that non-stepping balance recovery ability would be most sensitive to ankle plantar flexor strength, compared to hip extensor, hip flexor, or ankle dorsiflexor strength.

## Materials and methods

### Human subjects and experimental protocol

Two groups of adults completed the study, including five younger adults (mean ± standard deviation age 23.4 ± 2.5 years; height 1.74 ± 0.07 m, mass 72.5± 8.1 kg, body mass index 23.9 ± 1.9 kg/m^2^, two women) and six community-dwelling older adults (age 73.2 ± 2.2 years; height 1.65 ± 0.09 m, mass 68.3 ± 7.4 kg, body mass index 25.0 ± 1.9 kg/m^2^, three women). A Wilcoxon rank-sum test did not detect any differences between groups in height, mass, and BMI. All subjects were able to stand and walk unassisted, and self-reported no medical conditions that influenced their balance. The study was approved by Virginia Tech Institutional Review Board, and written consent was obtained from all subjects prior to participation.

Non-stepping balance recovery ability was operationalized by determining the maximal backward platform displacement from which subjects could recover balance without stepping. Displacements were induced using a 0.9 × 0.9 m, pneumatically-actuated, moving platform. Both backward and forward platform displacements were used to minimize the anticipation of the direction of platform displacement by the subjects. However, only backward displacement of the platform was used to determine maximal platform displacement because the focus of this study was to build upon prior work investigating the use of the ankle strategy to recover from forward falls [4–7]. The platform had the capability to translate 0–0.25 m backward and 0–0.15 m forward in approximately 350 ms.

### Balance perturbations

Subjects stood barefoot on the platform with their feet approximately shoulder width apart, eyes open, and looking straight ahead while keeping their arms crossed. Prior to any perturbation, subjects were instructed to remain relaxed, try not to step, and remain standing still after a perturbation. No restrictions were given on how to respond to the perturbations (i.e. subjects were allowed to bend their hip, if necessary or desired). An assistant stood nearby to provide assistance with recovering balance, if needed. In the first trial, the platform moved approximately 2 cm backward. If the trial was successful (i.e., non-stepping and without assistance) trial, a subsequent trial was performed with the displacement increased by 1 cm. If the trial was unsuccessful (i.e. the subject stepped or required assistance to maintain balance), another attempt was provided at the same displacement. If this second attempt was successful, the displacement was increased 1 cm and repeated. This process was repeated until three unsuccessful trials occurred at the same platform displacement. Both forward and backward platform translations were completed, using the same procedures, and were presented in random order to minimize anticipation of translation direction.

Prior to these trials, reflective markers were placed bilaterally at the acromion process, anterior superior iliac spine, posterior superior iliac spine, greater trochanter, lateral femoral epicondyle, lateral malleolus, calcaneous, and head of the 5^th^ metatarsal. During the perturbations, marker coordinates were sampled at 100 Hz using a 6-camera motion analysis system (MX-T10, Vicon Motion Systems Inc., Los Angeles, CA) and subsequently low-pass filtered at 40 Hz (4^th^-order Butterworth filter). Marker kinematics were used to estimate joint kinematics in the subsequent simulation study.

### Statistical analysis

Maximal backward platform displacement was compared between young and older groups using a Wilcoxon rank-sum test. A significance level of *p* < 0.05 was used, and the analysis was performed using JMP v10 (SAS Institute, Inc, Cary, NC).

### Simulations

Forward dynamic simulations were used to investigate the relative importance of lower limb muscle group strength on non-stepping balance recovery ability (i.e. maximal platform displacement). In step one of this study, the model was used to essentially repeat the human subjects experiment to investigate the effects of age-related strength loss on maximal platform displacement. This allowed a comparison of the reduction in maximal platform displacement between simulations and human subjects, and provided some validation for model performance. In step two of this study, the effects of reducing the strength of *individual* muscle groups was investigated to determine their relative effect on maximal platform displacement.

**Fig 1:**
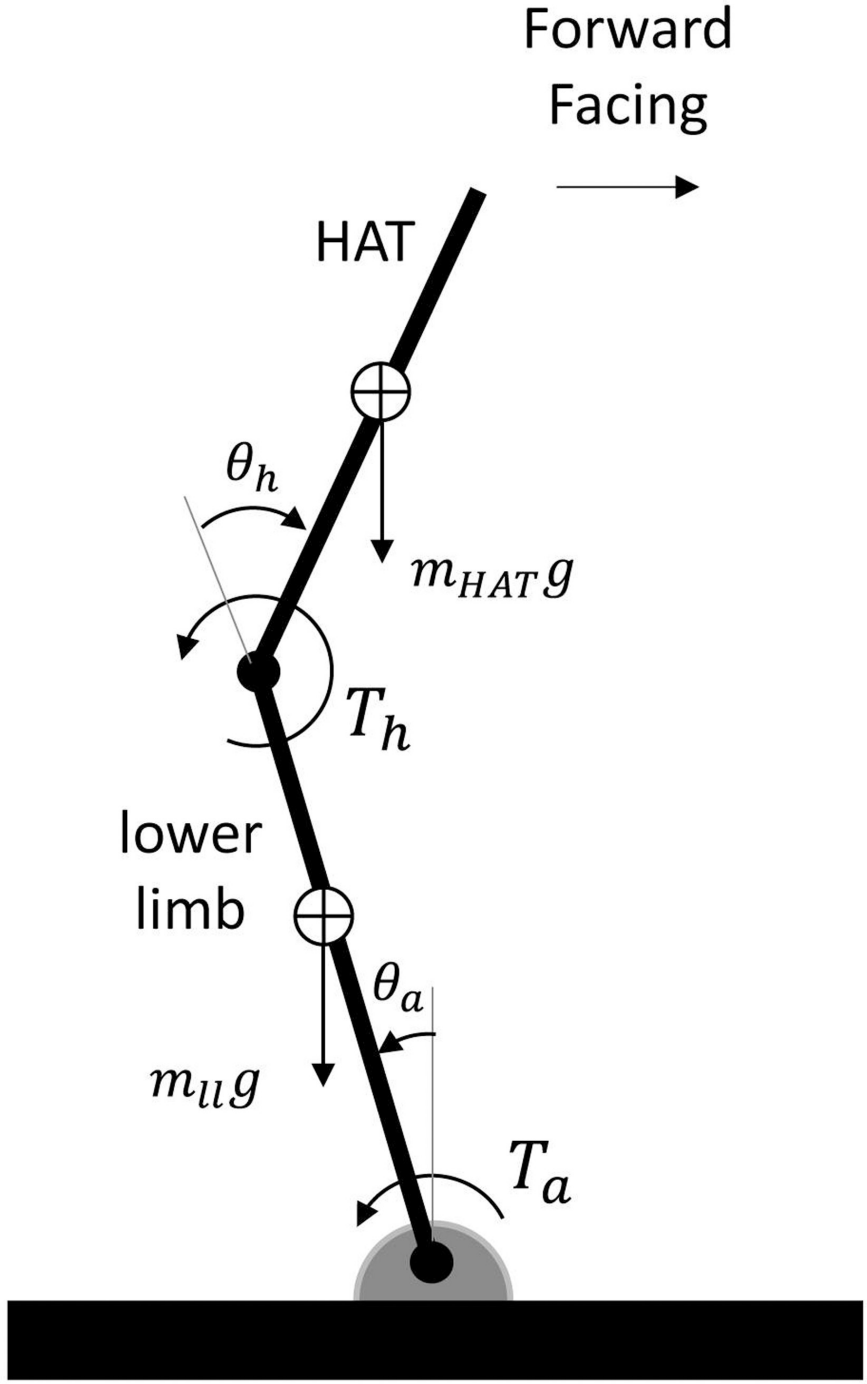
Diagram of the two-segment, rigid-body model where *θ*_*h*_ = hip angle, *θ*_*a*_ = ankle angle, *T*_*h*_ = hip torque, *T*_*a*_ = ankle torque, HAT = head, arms, and trunk segment, *m*_*ll*_ = mass of lower limb segment, *m*_*HAT*_ = mass of HAT segment, *g* = acceleration due to gravity.

The simulation model is briefly described here, with additional details provided in the Supporting Information S1 File. A sagittal plane, two-segment, torque-driven model of a representative young male subject (age 21 years, height 1.7 m, body mass 66.5 kg) was used. One segment represented the lower limbs (shank and thigh), and the other segment represented the HAT (head, arms, and trunk) segment (Fig 1). A knee joint was not included because only relatively small changes in knee angle (~ 5 deg) were observed during human subjects testing. A foot segment was also not included because only small heel rises (< 2.5 cm) were observed during human subjects testing. Instead, a pin joint, representing the ankle, connected the distal end of the lower limb to the floor. Segment mass and inertial parameters were estimated from de Leva [10], and doubled for the lower limb segment because this segment represented the left and right lower limbs. Torque actuators at the ankle and hip represented muscle actuation. The torque produced by these actuators was determined using a model that incorporated multiple aspects of muscle function. Each torque was the sum of a passive elastic torque, which was a function of angle and angular velocity [11], and an active torque, which was a function of maximum isometric torque [12], joint angle, joint angular velocity, and joint activation (an analog of muscle activation) [13–15]. The equations of motion were derived using Autolev software (OnLine Dynamics, Inc., Sunnyvale, CA), and integrated using a fixed-step-size (0.001 sec) 4^th^-order Runge-Kutta method.

Joint activation was an analog of muscle activation, and was applied to each torque actuator rather than an individual muscle. As such, it could elicit a joint torque ranging from the maximum extensor torque to the maximum flexor torque. Joint activation was defined from 19 joint activation “nodes” spaced at 50 ms increments over the 900 ms duration of each simulation, with the joint activation between nodes being determined using linear interpolation. Values of the joint activation nodes were determined using a simulated annealing optimization algorithm and a cost function that attempted to maintain standing balance after the perturbation. The cost function was developed based upon the work of Yang, Anderson [16], who used simulated annealing to elicit responses to a backward balance loss following a slip during gait.

**Fig 2:**
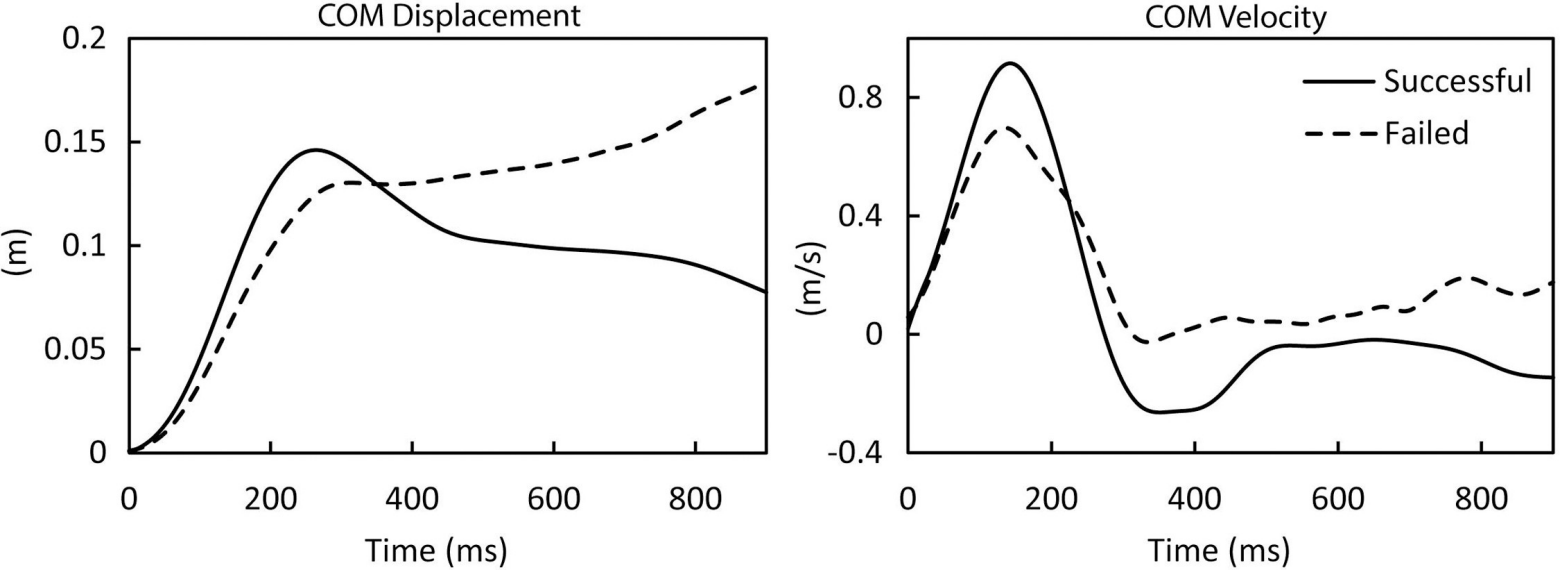
Whole-body COM displacement (left) and velocity (right) for representative trials from a young human subject. Positive COM displacement and COM velocity indicate anterior displacement from the ankle or anterior velocity.

Prior to initiating the simulation study, a scaling factor was determined to account for performance differences between the young model and the human subject whom the model represented [5]. The scaling factor, when multiplied by all joint activation values, resulted in the young model exhibiting the same maximal platform displacement as this human subject. Maximal platform displacement was first determined using a scaling factor of 1.0 using the following procedure. Starting with a platform displacement of 2 cm, the optimization was performed to determine if the model could successfully recover balance. Each simulation was deemed a successful recovery if the whole-body COM did not move anteriorly past the limit of the base of support (head of the 5th metatarsal), and the horizontal velocity of the whole-body COM became negative (posterior movement), and remained negative, for at least 500 ms. These criteria were selected based upon an investigation of the successful and failed recoveries in human subject testing (Fig 2). If these two criteria were not satisfied, then the trial was deemed a failure. After a successful recovery, the platform displacement was increased by 1 cm, and the optimization was repeated. This process was continued until the platform displacement was too large for the model/optimization to successfully recover balance. Maximal platform displacement was the largest backward displacement from which the model could successfully recover. The process of varying the scaling factor and determining the maximal platform displacement was then repeated to determine the scaling factor (0.57) that provided the same maximal platform displacement of the human subject whom the model represented (19 cm).

In step one of the simulation study, the maximum isometric torque, *T*_*max*_, of the two torque actuators in flexion and extension were simultaneously reduced to mimic the age-related loss of muscle strength. The reduction percentage was based upon the data of Harbo, Brincks [17], who reported age-related differences in lower limb strength between young men (15–30 years) and older men (70–80 years). Based on their data, *T*_*max*_ was reduced by 35% for hip extension (HE), 25% for hip flexion (HF), 35% for ankle plantar flexion (PF), and 16% for ankle dorsiflexion (DF). The reductions were made compared to the maximum isometric torque (*T*_*max*_) used for the torque actuators of the young model (HE 178 Nm, HF 125 Nm, PF 105 Nm, DF 36 Nm). Maximal platform displacement was then found with this “older model” using the same procedures described above.

In step two of the simulation study, the effects of *individually* reducing one of these four *T*_*max*_ values was investigated to determine their relative effect on maximal platform displacement. This involved eight different cases of age-related strength loss. In the first four cases (HE_35%_, HF_25%_, PF_35%_, DF_16%_), one *T*_*max*_ was reduced by the same percentage as strength loss percentages reported by Harbo, Brincks [17]. In the second four cases (HE_20%_, HF_20%_, PF_20%_, DF_20%_), one *T*_*max*_ was reduced by 20%. The reductions were made compared to the maximum isometric torque (*T*_*max*_) used for the torque actuators of the young model (HE 178 Nm, HF 125 Nm, PF 105 Nm, DF 36 Nm). In each case, the maximal platform displacement was found using the same procedure described above. These two methods of strength reduction were used to evaluate the relative effect of strength loss when considering typical reductions in associated with aging, and when considering equal reductions.

## Results

### Maximal platform displacement

**Fig 3:**
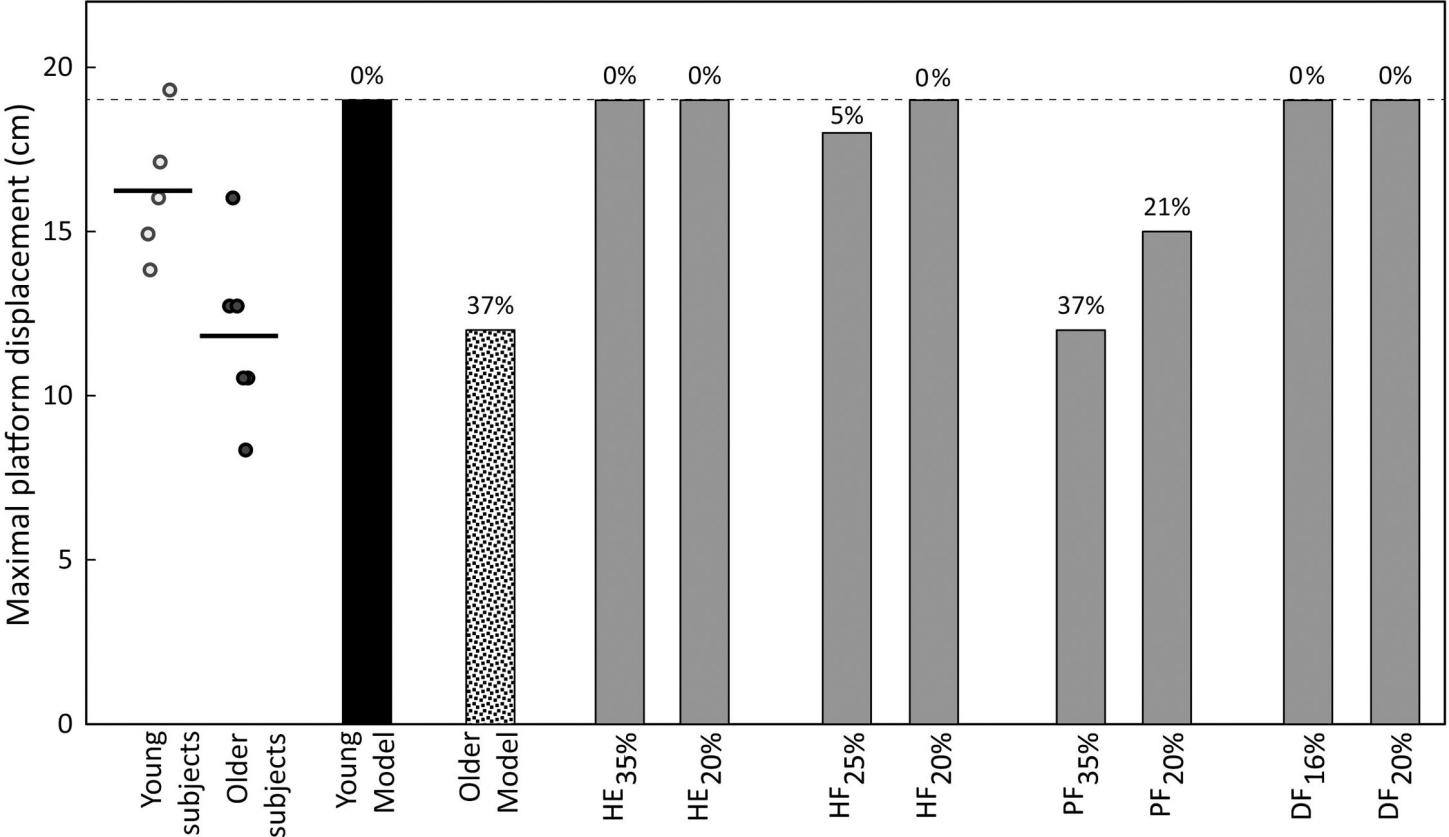
Maximal platform displacement for young and older human subjects (horizontal line indicates mean for each group), and for simulations of different cases of strength loss. Data labels represent the percent reduction in maximal platform displacement compared to the young model (19 cm).

During human subjects testing, maximal platform displacement was 27% smaller (*p* = 0.027) among the older group (mean ± standard deviation 11.8 ± 2.1 cm) compared to the young group (16.2 ± 2.6 cm). The maximum platform displacement of each subject is included in online Supporting Information S2 File. In step one of the simulation study, maximal platform displacement was 19 cm when using the young model (identical to the young human subject who was modeled), and decreased 37% to 12 cm when using the older model that included age-related strength loss in HE, HF, PF, and DF. In step two of the simulation study, maximal platform displacement was lower after some, but not all of the simulated individual strength losses. Compared to the maximal platform displacement value of 19 cm for the young model, maximal platform displacement was reduced for PF_35%_, PF_20%_, and HF_25%_, but not with HE_35%_, DF_16%_, HE_20%_, HF_20%_, PF_20%_, DF_20%_ (Fig 3).

### Simulation kinematics and torques

**Fig 4:**
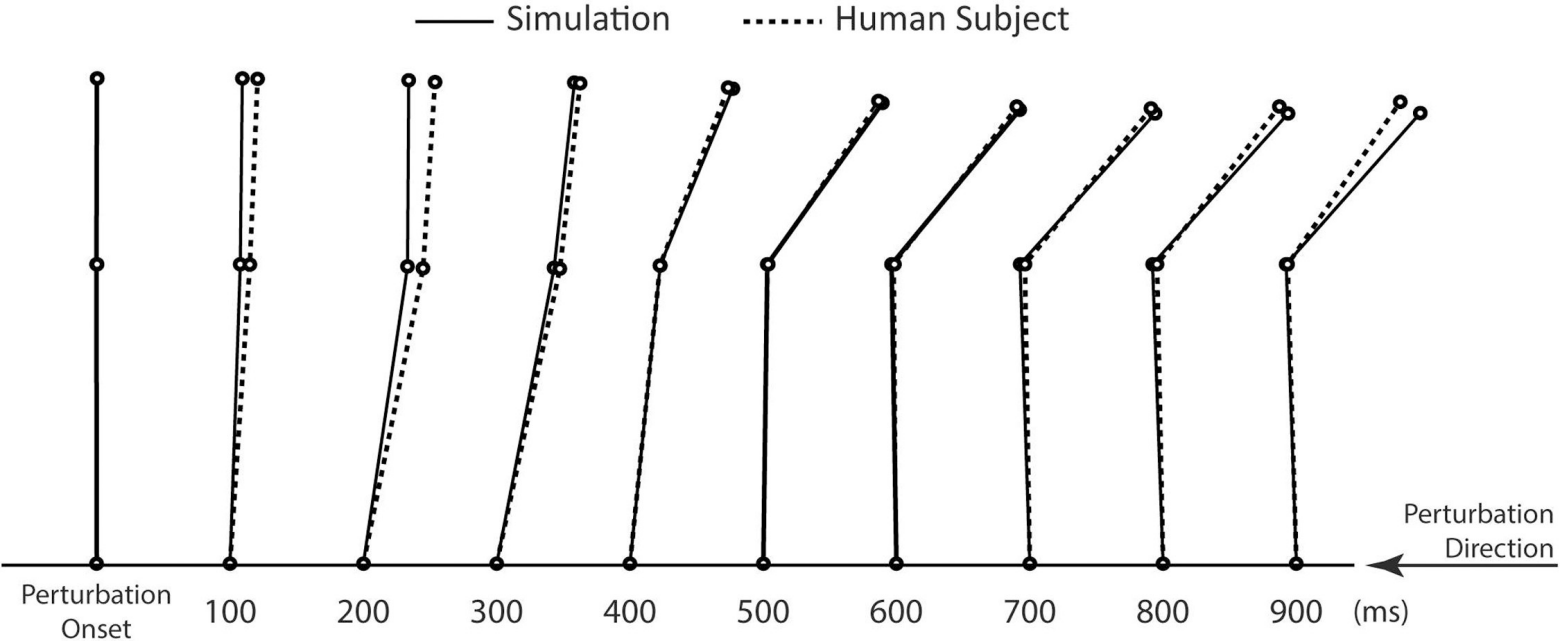
Comparison of body position between the human subject and simulation during the maximal platform displacement trial of the young model.

**Fig 5:**
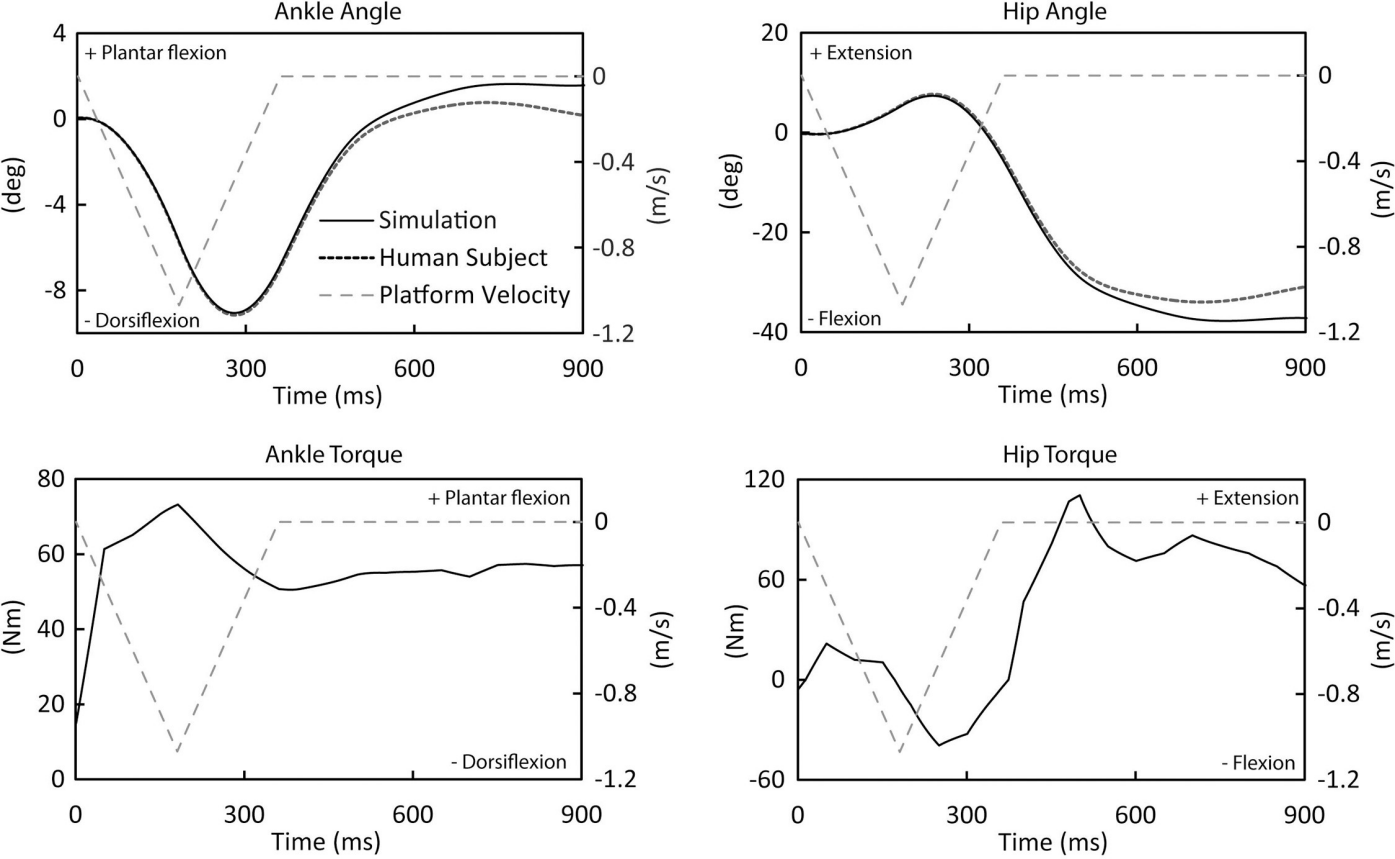
Joint angles, torques, and platform velocity (secondary *y*-axis) during the trial at maximal platform displacement of the young model. These results illustrate the general pattern of these variables over time during simulations, and similarities between ankle and hip angles between the simulations and human subjects testing.

Joint angles exhibited the same general pattern between simulations and the modeled subject (Figs 4 and 5). For example, during the maximal platform displacement trial prior to applying strength loss, the RMS joint angle differences were 1.0 degree at the ankle and 2.4 degrees at the hip. Joint torques exhibited a similar pattern across simulations. Ankle torque exhibited a peak in plantar flexion immediately following perturbation onset, and then a maintained plantar flexion dominance throughout the rest of the simulation. Hip torque initially exhibited a small peak in hip extension, followed by a peak in hip flexion, and then a larger peak in extension and extension dominance for the rest of the simulation.

**Fig 6:**
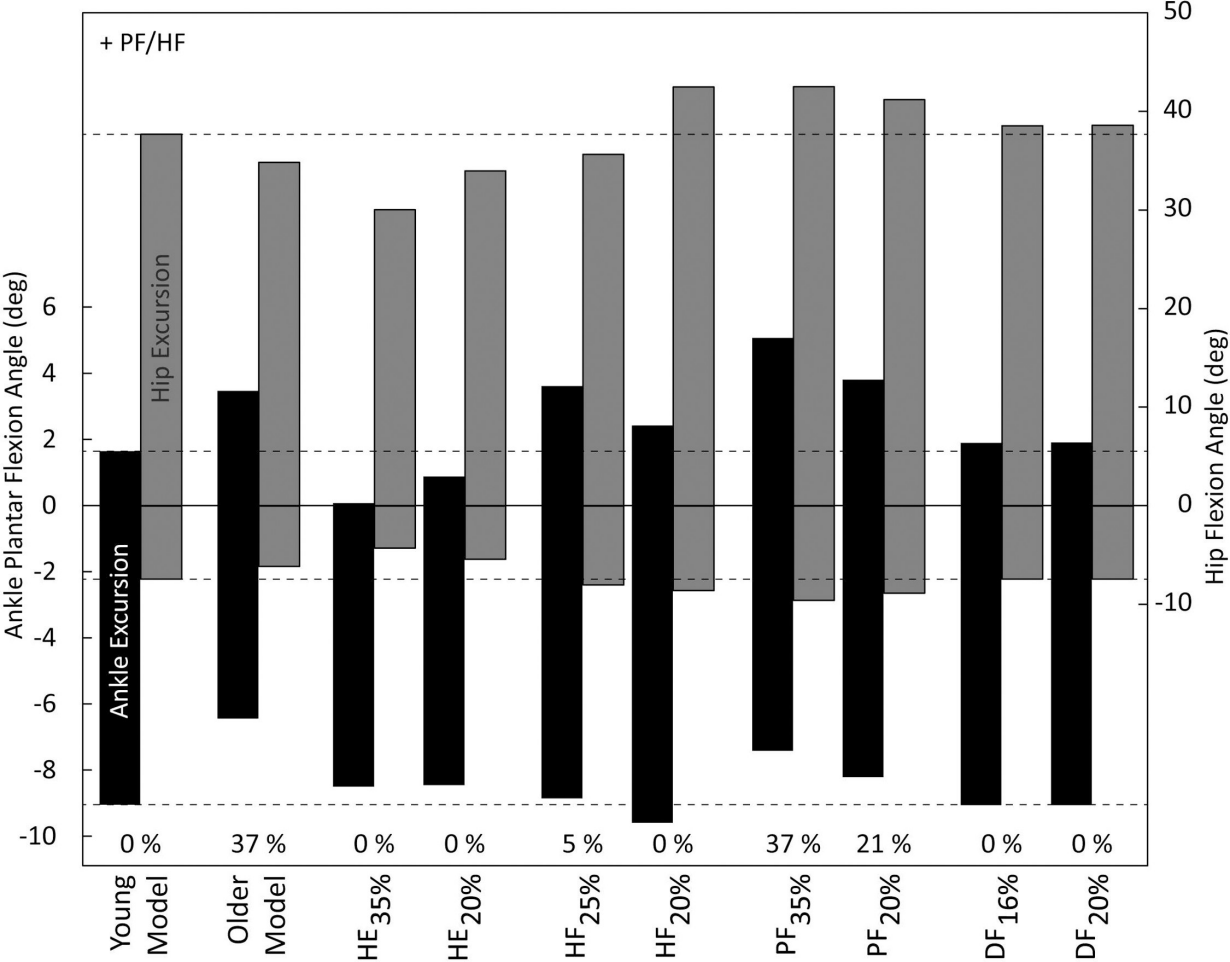
Peak joint angles at maximal platform displacement for young and older models, and all cases of strength loss. The top of the bars indicate peak ankle plantar flexion/hip flexion angle, and the bottom of the bars indicate peak dorsiflexion/hip extension angle. The total height of the bars indicate the range of ankle and hip joints used. Note the difference in range between the ankle (left) and hip (right) y-axes. The percentages at the bottom represent the percent reduction in maximal platform displacement compared to the young model.

**Fig 7:**
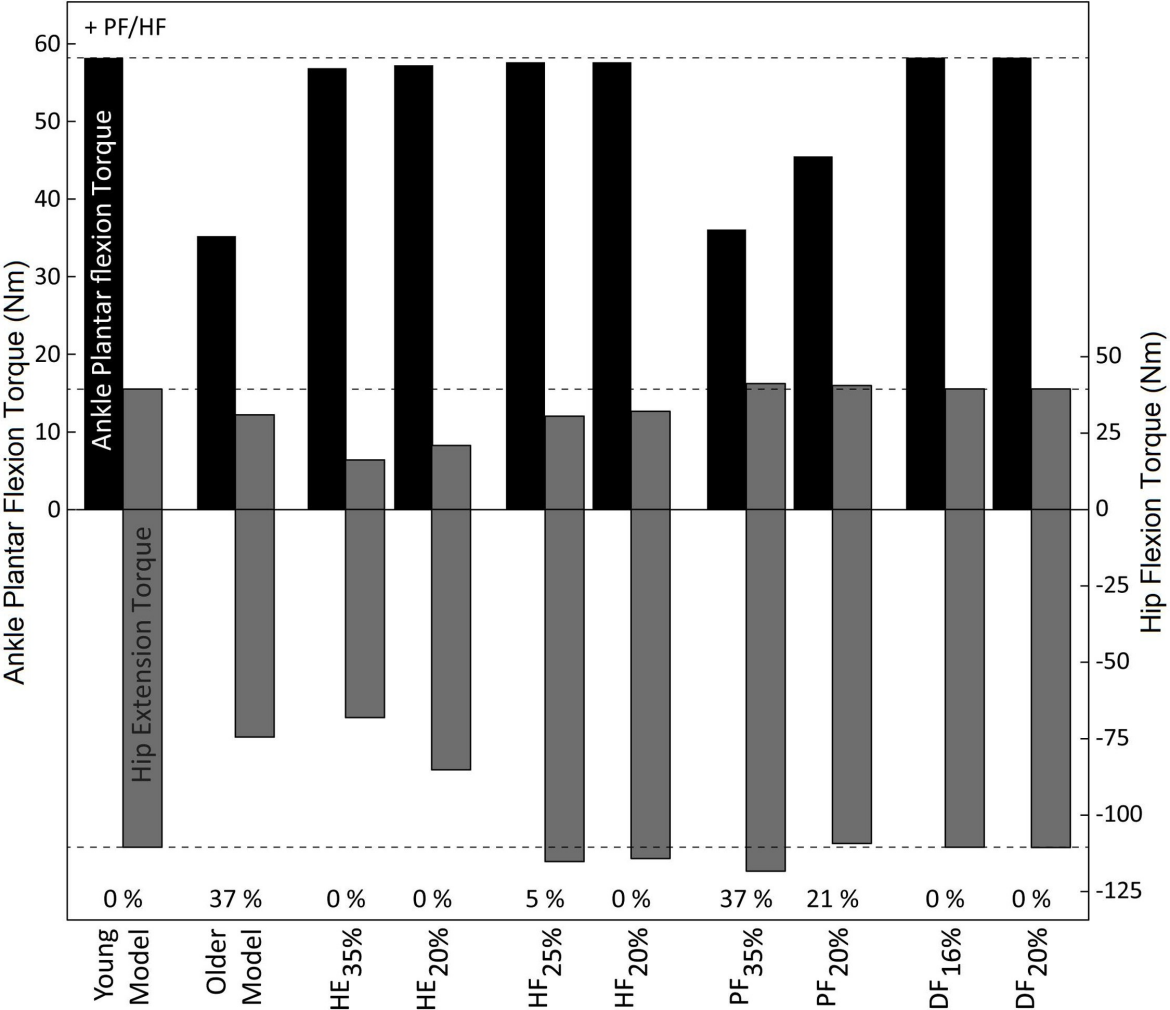
Peak joint torques at maximal platform displacement for young and older model, and all cases of strength loss. The top of the bars indicate peak ankle plantar flexion/hip flexion torque, and the bottom of the bars indicate peak dorsiflexion/hip extension torque. The percentages at the bottom represent the percent reduction in maximal platform displacement compared to the young model.

In addition to a decrease in maximal platform displacement, strength loss also resulted in changes in joint angles (Fig 6) and joint torques (Fig 7). In step one of the simulation study when using the older model, peak DF angle, peak PF torque, and peak HE torque were all smaller compared to balance recovery with the young model. In step two of the simulation study when investigating individual strength losses, peak joint angles and torques also changed relative to balance recovery with the young model, with notable changes summarized here. During *HE*_*35%*_ and *HE*_*20%*_ when maximal platform displacement did not differ from the young model, peak joint angles in HF, HE, DF, and PF were smaller, and peak torques in HE and HF were also smaller. During HF_25%_ and HF_20%_ when maximal platform displacement was 0–5% smaller compared to the young model, peak PF angle was larger, and peak HF torque was smaller. During PF_20%_ and PF_35%_ when maximal platform displacement was 21–37% smaller compared to the young model, peak HF and PF joint angles were larger, peak DF angle was smaller, and peak PF torque was smaller. During DF_16%_ and DF_20%_ when maximal platform displacement did not differ from the young model, peak joint angles and torques showed minimal changes compared to the young model.

## Discussion

The purpose of this study was to investigate the effects of age-related strength loss on non-stepping balance recovery after a perturbation, without constraining movements to an ankle strategy as had been done in earlier work. Our first hypothesis was that older adults would exhibit a reduced non-stepping balance recovery ability compared to young adults. This was supported given the 27% smaller maximal platform displacement among older adults. This age-related reduction was slightly lower than an earlier-reported 36% reduction in maximum lean angle from which subjects recovered using an ankle strategy [4]. The smaller age-related difference in non-stepping balance recovery observed here may be a result of allowing for the use of hip motion because older adults rely more on a hip strategy to recover balance [18, 19]. Moreover, constraining recovery movement to an ankle strategy may make standing balance more dependent upon PF strength. As PF strength declines substantially with age [17, 20], this constraint can be expected to have a large impact on older adults. Another difference between the current study and prior work is that perturbations used here involved translation of a support surface, while prior work released participants from a static forward lean angle. These may have resulted in different sensory stimuli at perturbation onset [21], and differences in the movements required for recovery [22] that may be differentially influenced by aging.

Subsequently, we used forward-dynamic simulations to investigate the influence of strength loss on non-stepping balance recovery. When muscle strength at all joints was reduced concurrently to simulate age-related strength loss, the model predicted a 37% reduction in maximal platform displacement, in relatively good agreement with the 27% reduction found among human subjects. While this provides some validation for the model and optimization approach, several simplifications of our modeling approach were notable. First, simulations were performed using a torque-driven model that did not allow for manipulation of specific muscles. While muscle-level actuators would allow for consideration of bi-articular muscles and muscle co-contraction, previous work has found good agreement between human subject and model kinematics using torque-driven models [13, 23]. Second, the model was only two-dimensional, and therefore neglected movements and any potentially relevant muscle forces that acted outside of the sagittal plane. However, this assumption was deemed reasonable given the lack of substantial asymmetry in movements during human subjects testing. Third, the model did not include a foot segment and knee joint, based upon comparatively small heel rises and knee angular displacements during human subjects testing. Prior simulation studies of ankle and hip strategies in response to postural perturbations while standing have used similar simplifying assumptions [9, 24]. We acknowledge these assumptions may have influenced our quantitative results, but do not suspect a meaningful influence on our overall findings. Finally, we only focused on age-related loss of muscle strength, yet the age-related difference in maximal platform displacement among human subjects may been influenced by other factors not modeled here such as reaction time, torque development rate, or standing balance.

Other limitations of our human subjects testing were also noteworthy. The platform did make an audible ‘click’ when the air valve opened at the onset of all perturbations. However, this click was virtually coincident with the onset of movement of the platform, and thus we do not think subjects were able to use this noise as warning of an impending perturbation. Our choice of a 1 cm increment in platform displacement was based upon our pilot testing and three reasons. First, it was sufficiently small to characterize the variability of maximal displacement among our subjects. Second, it was sufficiently small to allow numerous trials to prior to reaching the maximal displacement, so that adaptation of performance to repeated perturbations could occur. We wanted our measurement of maximal displacement to occur after adaptation so that our results were more reliable. Third, it was not so small such that too many trials would be required to reach the maximal displacement, and thus make the protocol fatiguing or too time-consuming. Using a larger increment may not have allowed us to accomplish all three of these goals.

When reducing strength at each joint individually, the relatively large reductions in maximal platform displacement predicted with PF_35%_ (37%) and PF_20%_ (21%) support earlier evidence of the importance of ankle strength in non-stepping balance recovery [4, 5, 25], even when movement is not constrained to the ankle strategy. These reductions in maximal platform displacement were larger than when reducing strength in HE, HF, and DF by either an amount that approximates age-related strength loss, or by a fixed 20%. In fact, reducing PF strength individually by 35% resulted in the same reduction in maximal platform displacement as when reducing strength at all four joint/exertions directions (see older model in Fig 3). The 21% reduction in maximal platform displacement for PF_20%_ was in good agreement with a reported 19% reduction in maximum recoverable lean angle with a 20% decrease of isometric ankle plantar flexor force using a single-segment model with a pure ankle strategy [5]. Consistent with the findings here, middle-aged patients with distal muscle weakness of trunk, arms, and legs (limb girdle muscular dystrophy) exhibited greater difficulty in non-stepping balance recovering after a backward tilt of a support surface, compared to patients with proximal muscle weakness of trunk, arms, and legs (distal spinal muscular atrophy) [26]. After PF, maximal platform displacement was most sensitive to HF strength loss (although only a minimal amount for HF_25%_ and not for HF_20%_). The small reduction in maximal platform displacement in the cases of HF strength loss (5% for HF_25%_ and 0% for HF_20%_) was likely due to 21% lower peak in hip flexion torque, since minimal differences in peak ankle plantar flexion and hip extension torque were observed compared to the young model.

In the case of PF_35%_, the model predicted 24% higher peak in hip flexion torque and 57% higher peak in hip extension torque compared to the older model which yielded a similar maximal platform displacement. Moreover, in case of PF_35%_, joint excursion was 20% higher at the ankle and 27% higher at the hip compared to the older model. The higher peak torques at the hip and higher hip excursion with PF_35%_ suggest a greater reliance on the hip during balance recovery compared to the older model, but also that this greater reliance was insufficient to maintain the same maximal platform displacement as in the young model. The higher observed ankle excursion may be due to the higher hip excursion while recovering balance, which moved the lower limb backward and consequently increased the ankle excursion [9]. The higher peak in hip extension torque may have been generated to compensate for higher peak in hip flexion.

The lack of difference in maximal platform displacement predicted with HE_35%_, HE_20%_, DF_16%_, DF_20%_ and the young model suggests a smaller influence of these muscle groups on non-stepping balance recovery capability. Although the hip extensors were responsible for decelerating the forward movement of HAT, and bringing the model to the upright position once the perturbation stopped, balance recovery capability was mostly influenced by the magnitude of ankle plantar flexor torque early in the recovery phase. This helps explain why a reduction in hip extensor strength did not have a strong influence on maximal platform displacement. When comparing HE_35%_, HE_20%_ to the young model, lower peak in hip extension and hip flexion torques were observed with HE strength reduction, which resulted in lower hip excursion and consequently lower ankle excursion (a more erect posture) during balance recovery. It may be that the model did not have sufficient hip extensor strength to compensate for the potentially higher magnitude of hip excursions, instead recovering with smaller hip excursion. Supporting this speculation, the magnitude of hip excursion was reduced less with HE_20%_ (20%) compared to HE_35%_ (24%). The lack of an effect of dorsiflexion strength reduction on maximal platform displacement was mainly due to a small involvement in non-stepping balance recovery. When comparing DF_16%_ and DF_20%_ to the young model, the peak joint torques and joint excursions did not differ.

## Conclusions

Non-stepping balance recovery ability was reduced among older adults when the movement was not constrained to an ankle strategy, similar to earlier work that used this constraint. Simulation results suggest that plantar flexor strength plays a major role in the capability to recover balance, even when the movement is not constrained to the ankle. Hip flexor muscle strength also appears to influence balance recovery capability when weakened, but the magnitude of this effect was smaller than that of ankle plantar flexor strength. A loss of hip extension and ankle dorsiflexion strength did not influence balance recovery. These findings improve our understanding of the importance of age-related strength loss on non-stepping balance recovery capability, and provide evidence of the relative contribution of the individual lower extremity muscle groups.

## Supporting information

S1 File Supporting information. (DOCX)

S2 File Supporting information excel file. (XLSX)
