# Supplementary material for: Age-related strength loss affects non-stepping balance recovery
Source: PLoS One. 2019 Jan 18;14(1):e0210049. doi: 10.1371/journal.pone.0210049 (PMC6338353; doi:10.1371/journal.pone.0210049)
Supplement: S1 File — (DOCX) [file pone.0210049.s001.docx]

**Supporting information**

For the two segment model used in the simulation study, torques produced by the ankle and hip actuators were determined by incorporating multiple aspects of muscle function. Each torque was the sum of a passive elastic torque, which was a function of angle and angular velocity [[1](#_ENREF_1)], and an active torque, which was a function of maximum isometric torque [[2](#_ENREF_2)], joint angle, joint angular velocity, and joint activation [[3-5](#_ENREF_3)]:

$$T_{passive}= f\left( \theta, \dot{\theta} \right)$$

$$T_{active}= T_{max} \times f\left( \theta\right)\times h\left( \omega\right)\times a(t)$$

$$T=T_{active}+T_{passive}$$

Where *T_max_* is the maximum isometric torque (Nm); *f(θ)* and *h(ω)* are dimensionless scaling factors for angle and angular velocity ranging from 0 to 1; and *a(t)* is the joint activation that varied from -1 to 1 to represent maximum activation of the flexors and extensors, respectively. Specific *T_max_* values for the young model were obtained from previous data [[2](#_ENREF_2)]. The torque-angle relation, *f(θ)*, was found using a polynomial fit of existing experimental data [[6](#_ENREF_6)]. The torque-angular velocity relation, *h(ω)*, was defined as:

$$\left\{ \begin{aligned} h\left( \omega\right)=\frac{(\omega_{0}-\omega)}{(\omega_{0}- \Gamma\omega)} , \frac{\omega}{\omega_{0}}<1 \\ h\left( \omega\right)=0 ,\frac{\omega}{\omega_{0}}\geq1 \end{aligned} \right.$$

where ω is the instantaneous angular velocity, ω_0_ is the maximum angular velocity, and Г is the shape factor of the torque-angular velocity curve [[5](#_ENREF_5), [7](#_ENREF_7)]. Joint activation, *a(t)*, is used as an analog of muscle activation, and could elicit a joint torque ranging from the maximal extensor torque to the maximal flexor torque. Joint activation was defined from 19 joint activation “nodes” spaced at 50 msec increments over the 900 ms duration of each simulation, with the joint activation between nodes being determined using linear interpolation (Figure A).


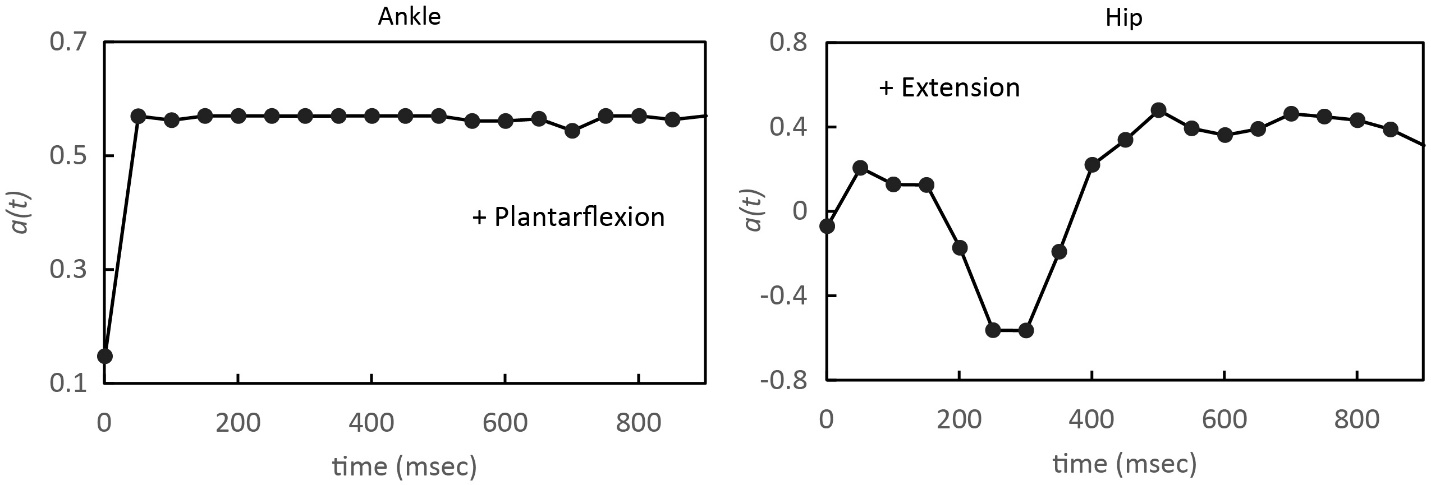


**Figure A.** Sample joint activations of the ankle (left) and hip (right) actuators for a representative simulation (displacement = 19 cm). Black dots represent joint activation nodes, while the connecting lines illustrate the linear interpolation between nodes. Time = 0 indicates the start of platform displacement.

Values of the joint activation nodes were determined using a simulated annealing optimization algorithm, and a cost function based upon model kinematics that promoted a non-stepping balance recovery response. The cost function was developed based upon the work of [Yang, Anderson [8](#_ENREF_8)], who used simulated annealing to elicit responses to a backward balance loss following a slip during gait. The cost function was defined as:

$$f=w_{1}\int_{t_{s}}^{t_{f}} \left| X_{COM}-X_{ankle} \right|dt+ w_{2}\int_{t_{s}}^{t_{f}} \left| \dot{X}_{COM}-\dot{X}_{ankle} \right|dt+ w_{3}\int_{t_{i}}^{t_{f}} e(\theta\left( t \right))dt+ w_{4}\int_{t_{i}}^{t_{f}} e(\dot{\theta}\left( t \right))dt+ w_{5}\sqrt{\sum_{i=1}^{2} {\dot{q_{i}}}^{2}}$$

where *t_i_* and *t_f_*, indicate the start and end times of the simulation, respectively. We set *t_i_* = 0 and *t_f_* = 900 msec based upon the human subjects data that indicated this duration was sufficient to determine the outcome (success or failure) of each trial. The first and second terms in the cost function were used to minimize horizontal whole-body COM displacement (*X_COM_*) relative to the ankle (*X_ankle_*), and the horizontal whole-body COM velocity $\dot{(X}_{COM})$ relative to the ankle ($\dot{X}_{ankle})$, respectively. This minimization was performed starting from *t_s_* that represented an activation delay, and was set to 150 msec [[9](#_ENREF_9)]. The third and fourth terms restricted joint angles and angular velocities to within physiological limits [[10](#_ENREF_10)], where $e\left( \theta\left( t \right) \right)=\sum\varphi(s_{i}\left( t \right))$ and $\varphi\left( s_{i}\left( t \right) \right)=\left\{ \begin{aligned} s_{i}^{-}-s_{i} s_{i}<s_{i}^{-} \\ 0 {s_{i}^{-}<s}_{i}< s_{i}^{+} \\ s_{i}-s_{i}^{+} s_{i}>s_{i}^{+} \end{aligned} \right.$ , and $s_{i}^{-},$ and $s_{i}^{+}$ represented the upper and lower physical bound of joint angle and angular velocity [[8](#_ENREF_8)]. The fifth term minimized the square root of segments angular velocities ($\dot{q}),$ and was used to bring the model to rest at the end of the simulation. Weighting of each term, *w*, were determined by trial and error to make the model better simulate the human subject kinematics. The final values of the weights were *w_1_*=1, *w_2_*=0.1, *w_3_*=10, *w_4_*=1, *w_5_*=0.1 and were kept constant across all subjects and trials. Simulations were terminated when changes in consecutive objective function values did not exceed 0.1 for 20 iterations.

The platform motion during human subjects testing (Figure B) was used to model platform motion during simulations (Figure C). To construct the platform velocity profile of the model, the time at the end of the movement, the time at the peak velocity, and the peak velocity were chosen from the three trials and then a were incorporated in a linear extrapolation to be able to enter the desired peak displacement and thus the peak velocity and get the velocity profile as an output.


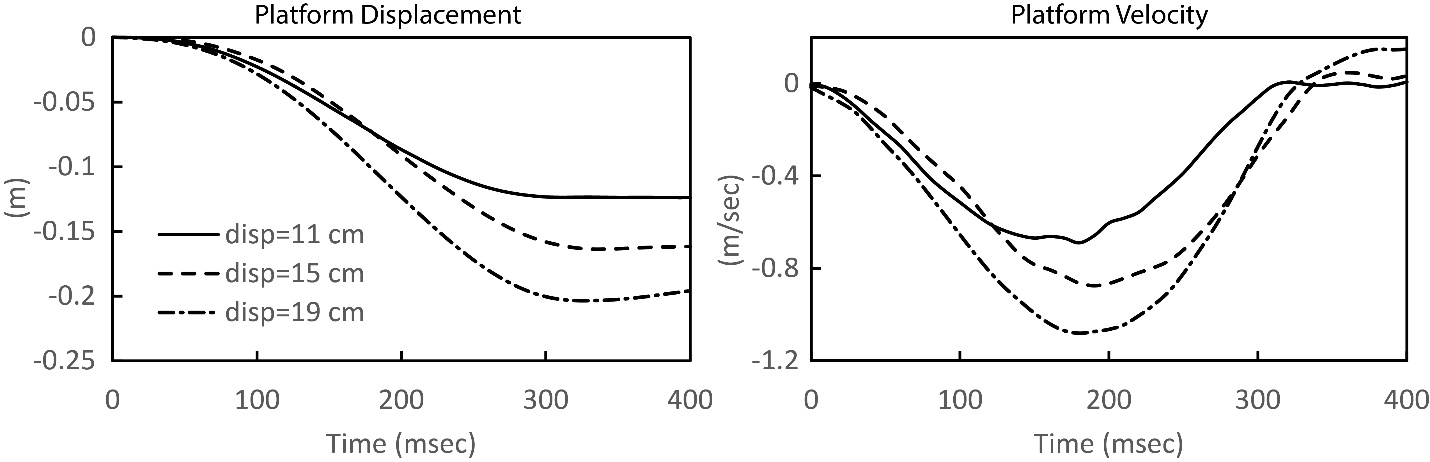


**Figure B.** Platform motion during human subjects testing, including displacement (left) and velocity (right) for three different perturbations (11 cm, 15 cm, and 19 cm). Displacement of the platform was varied by modulating the duration that a pneumatic solenoid was open (increasing this during increased the speed of platform movement). Perturbation durations were ~350 ms for all displacements.


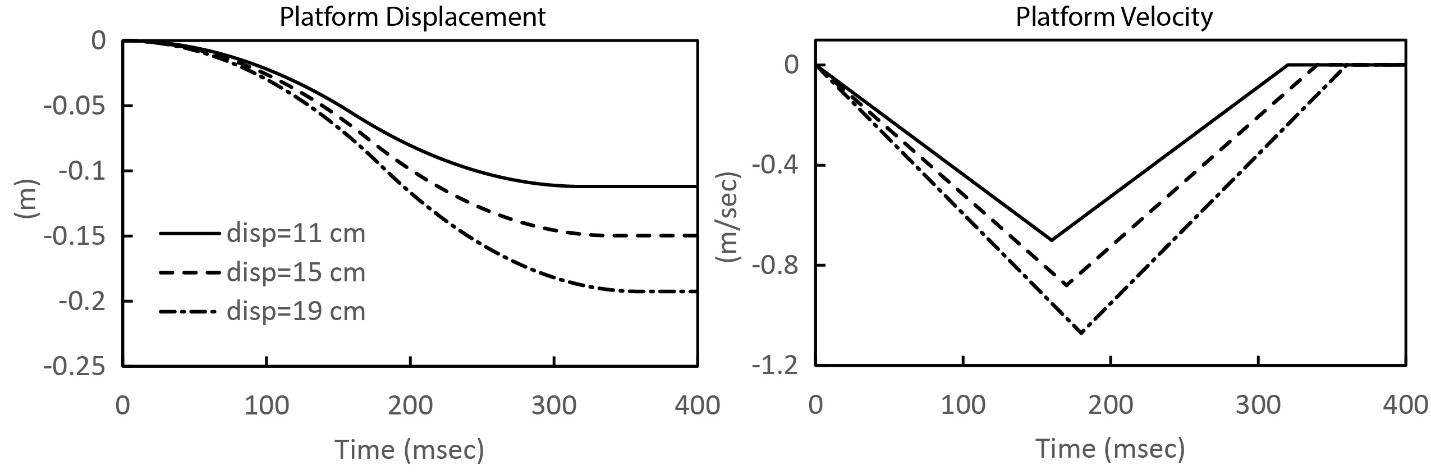


**Figure C.** Platform motion during the simulation study, including displacement (left) and velocity (right) profiles used to model three different perturbation magnitudes. Negative indicates backward.

**References**

1. Riener R, Edrich T. Identification of passive elastic joint moments in the lower extremities. Journal of Biomechanics. 1999;32(5):539-44. doi: <http://dx.doi.org/10.1016/S0021-9290(99)00009-3>.

2. Anderson DE, Madigan ML, Nussbaum MA. Maximum voluntary joint torque as a function of joint angle and angular velocity: model development and application to the lower limb. Journal of biomechanics. 2007;40(14):3105-13.

3. Ashby BM, Delp SL. Optimal control simulations reveal mechanisms by which arm movement improves standing long jump performance. Journal of Biomechanics. 2006;39(9):1726-34. doi: <http://dx.doi.org/10.1016/j.jbiomech.2005.04.017>.

4. Cheng KB, Wang C-H, Chen H-C, Wu C-D, Chiu H-T. The mechanisms that enable arm motion to enhance vertical jump performance—A simulation study. Journal of Biomechanics. 2008;41(9):1847-54. doi: <http://dx.doi.org/10.1016/j.jbiomech.2008.04.004>.

5. Selbie WS, Caldwell GE. A simulation study of vertical jumping from different starting postures. Journal of Biomechanics. 1996;29(9):1137-46. doi: <http://dx.doi.org/10.1016/0021-9290(96)00030-9>.

6. Hoy MG, Zajac FE, Gordon ME. A musculoskeletal model of the human lower extremity: The effect of muscle, tendon, and moment arm on the moment-angle relationship of musculotendon actuators at the hip, knee, and ankle. Journal of Biomechanics. 1990;23(2):157-69. doi: <http://dx.doi.org/10.1016/0021-9290(90)90349-8>.

7. Alexander RM. Sequential joint extension in jumping: Reaction to G.J. van Ingen Schenau (1989). Human Movement Science. 1989;8(4):339-45. doi: <http://dx.doi.org/10.1016/0167-9457(89)90038-9>.

8. Yang F, Anderson FC, Pai Y-C. Predicted threshold against backward balance loss following a slip in gait. Journal of biomechanics. 2008;41(9):1823-31.

9. Runge C, Shupert C, Horak F, Zajac F. Ankle and hip postural strategies defined by joint torques. Gait & posture. 1999;10(2):161-70.

10. Luttgens K, Wells KF. Kinesiology: scientific basis of human motion: Saunders College Pub.; 1982.
